# Supplementary material for: The prevalence of Schistosoma mansoni infection among adults with chronic non-communicable diseases in Malawi
Source: Trop Med Health. 2022 Aug 19;50:56. doi: 10.1186/s41182-022-00450-3 (PMC9389769; doi:10.1186/s41182-022-00450-3)
Supplement: Supplementary file 1 — Additional file 1: Comparison of participants who submitted and did not submit stool and urine samples. [file 41182_2022_450_MOESM1_ESM.docx]

Additional Table S1. Comparison of participants who submitted and did not submit stool and urine samples

|  | Submitted stool and urine specimens | | Did not submit stool and urine specimens | |
| --- | --- | --- | --- | --- |
| Total sample size (414) | N = 339 | | N = 75 | |
|  | Male | Female | Male | Female |
| N (%) | 137 (33%) | 277 (67%) | 38 (51%) | 37 (49%) |
| Comparison of socio-demographic characteristics | | | | |
| Age   - Mean (SD) | 55 (18) | 58 (14) | 58 (20) | 54 (13) |
| Education   - None - Less than primary - Primary school completed - Secondary school completed - College/university completed - Post graduate degree | 21 (15%)  58 (42%)  21 (15%)  24 (18%)  8 (6%)  5 (4%) | 67 (24%)  140 (51%)  35 (13%)  32 (12%)  3 (1%) | 8 (21%)  13 (34%)  7 (18%)  7 (18%)  2 (5%)  1 (3%) | 9 (24%)  16 (43%)  5 (14%)  6 (16%)  1 (3%) |
| Marital status   - Never married - Currently married - Separated - Divorced - Widowed - Cohabiting | 11 (8%)  107 (78%)  3 (2%)  5 (4%)  11 (8%) | 7 (3%)  141 (51%)  20 (7%)  26 (9%)  83 (30%) | 4 (11%)  27 (71%)  1 (3%)  1 (3%)  5 (13%) | 2 (5%)  20 (54%)  3 (8%)  4 (11%)  8 (22%) |
| Work status   - Government employee - Non – government employee - Self employed - Non – paid worker - Student - Home maker - Retired with benefits - Unemployed | 11 (8%)  9 (7%)  61 (45%)  8 (6%)  1 (1%)  3 (2%)  13 (9%)  31 (23%) | 12 (4%)  7 (3%)  81 (29%)  21 (8%)  15 (5%)  5 (2%)  136 (49%) | 2 (5%)  4 (11%)  16 (42%)  4 (11%)  1 (3%)  4 (11%)  7 (18%) | 2 (5%)  1 (3%)  11 (30%)  6 (16%)  3 (8%)  3 (8%)  11 (30%) |
| Average household income   - Mean (SD) | 87 016.15 (84 493.59) | 69 117.52 (86 475.66) | 81 113.89 (67 342.91) | 63 654.05 (54 697.40) |
| Comparison of selected behavioral risk factors and medical history | | | | |
| Smoking   - Current smoker - Ever smoked | 0  13 (9%) | 0  5 (2%) | 0  2 (5%) | 0  0 |
| Alcohol consumption   - Ever consumed - In the past 12 months - Stopped for health reasons | 12 (9%)  8 (6%)  8 (6%) | 4 (1%)  1 (0.4%)  2 (0.7%) | 3 (8%)  3 (8%)  1 (3%) | 0  0  0 |
| Cardiovascular disease   - Previous heart attack, chest pain (angina) or stroke - Currently taking aspirin to prevent or treat disease - Currently taking regular statins to prevent or treat | 8 (6%)  33 (24%)  24 (18%) | 13 (5%)  82 (30%)  54 (20%) | 2 (5%)  7 (18%)  7 (18%) | 2 (5%)  9 (25%)  6 (16%) |
| Comparison of biological risk factors and anthropometry | | | | |
| Body weight (kg)   - Mean (SD) | 64 (14) | 64 (18) | 61 (14) | 64 (23) |
| Blood pressure (mm/Hg)   - Systolic Mean (SD) - Diastolic Mean (SD) | 150 (34)  84 (17) | 156 (33)  88 (17) | 151 (39)  82 (17) | 150 (32)  85 (15) |
| Fasting blood glucose (mg/dl)   - Mean (SD) | 226 (114) | 250 (138) | 213 (105) | 292 (157) |
| Comparison of prevalence of NCD syndromes | | | | |
| Hypertension, % (95% CI) | 77% (69 – 83) | 90% (85 – 93) | 82% (66 – 92) | 84% (68 – 94) |
| Diabetes, % (95% CI) | 59% (50 – 67) | 33% (27 – 39) | 61% (43 – 76) | 35% (20 – 53) |
| Heart disease, % (95% CI) | 4% (1 – 8) | 3% (1 – 5) | 5% (0.7 – 18) | 5% (0.7 – 18) |
