# Supplementary material for: The prevalence of Schistosoma mansoni infection among adults with chronic non-communicable diseases in Malawi
Source: Trop Med Health. 2022 Aug 19;50:56. doi: 10.1186/s41182-022-00450-3 (PMC9389769; doi:10.1186/s41182-022-00450-3)
Supplement: Supplementary file 2 — Additional file 2: Behavioural risk factors and medical history of male and female participants. [file 41182_2022_450_MOESM2_ESM.docx]

Additional Table S2. Behavioural risk factors and medical history of male and female participants

|  | Male | Female | Difference in proportions (95% CI) | P - value |
| --- | --- | --- | --- | --- |
| Smoking   - Current smoker - Ever smoked | 0  13 (9%) | 0  5 (2%) | 7% (- 13 – 27) | 0.60 |
| Alcohol consumption   - Ever consumed - In the past 12 months - Stopped for health reasons | 12 (9%)  8 (6%)  8 (6%) | 4 (1%)  1 (0.4%)  2 (0.7%) | 8% (-11 – 27)  5.6% (-149 – 26)  5.3% (-148 – 25) | 0.59  0.81  0.76 |
| Raised blood pressure   - Told by doctor in the past 12 months - Taken medications for treatment in past 2 weeks | 115 (84%)  20 (15%) | 260 (94%)  36 (13%) | 10% (3 – 17)  2% (-17 – 21) | <0.01  0.83 |
| Diabetes   - Told by doctor in the past 12 months - Taken medications for treatment in past 2 weeks | 84 (62%)  74 (54%) | 91 (33%)  88 (32%) | 29% (15 – 43)  22% (7 – 37) | <0.001  <0.01 |
| Raised cholesterol   - Told by doctor in the past 12 months - Taken medications for treatment in past 2 weeks | 5 (4%)  2 (1%) | 17 (6%)  3 (1%) | 2% (-23 – 19)  0 (-10 – 12) | 0.86  1.00 |
| Cardiovascular disease   - Previous heart attack, chest pain (angina) or stroke (CVA) - Currently taking aspirin to prevent or treat heart disease - Currently taking regular statins to prevent or treat heart disease | 8 (6%)  33 (24%)  24 (18%) | 13 (5%)  82 (30%)  54 (20%) | 1% (-19 – 21)  6% (-24 – 12)  2% (-21 – 17) | 0.92  0.52  0.84 |
| Chronic kidney disease   - Ever been told by doctor - Ever been on dialysis or had kidney transplant | 1 (0.7%)  0 | 4 (1%)  1 (0.3%) | 0.3%  0.3% | -  - |
