# Supplementary material for: The prevalence of Schistosoma mansoni infection among adults with chronic non-communicable diseases in Malawi
Source: Trop Med Health. 2022 Aug 19;50:56. doi: 10.1186/s41182-022-00450-3 (PMC9389769; doi:10.1186/s41182-022-00450-3)
Supplement: Supplementary file 3 — Additional file 3: Regression analysis evaluating risk factors of hypertension. [file 41182_2022_450_MOESM3_ESM.docx]

Additional Table S3. Regression analysis evaluating risk factors of hypertension

| Variable | N (%) | Crude OR  (95% CI) | P – value | Adjusted OR  (95% CI) | P – value |
| --- | --- | --- | --- | --- | --- |
| S. mansoni  - Positive | 50 (15%) | 1.2 (0.5 – 3.1) | 0.47 | - | - |
| Age |  | 1.1 (1.05 – 1.09) | <0.001 | 1.1 (1.05 – 1.12) | <0.001* |
| Sex   - Male - Female | 137 (33%)  277 (67%) | 1  2.6 (1.5 – 4.5) | <0.01 | 2.1 (0.96 – 4.6) | 0.06 |
| Education   - None - Less than primary - Primary school completed - Secondary school completed - College/University completed - Postgraduate degree | 88 (21%)  198 (48%)  56 (14%)  56 (14%)  11 (3%)  1 (1%) | 1  0.2 (0.1 – 0.8)   - 1. (0.03 – 0.5)   2. (0.02 – 0.3)   3. (0.01 – 0.3)   4. (0.01 – 1.7) | -  0.02  <0.01  <0.001  <0.01  0.12 | -  0.7 (0.2 – 2.6)  0.2 (0.05 – 0.98)  0.4 (0.1 – 2.0)  0.8 (0.1 – 7.5)  - | -  0.55  0.05*  0.28  0.82  - |
| Marital status   - Never married - Currently married - Separated - Divorced - Widowed | 18 (4%)  248 (60%)  23 (6%)  31 (7%)  94 (23%) | 1  3.9 (1.5 – 10.5)  8.4 (1.5 – 47)  3.1 (1.1 – 15.8)  18 (4.6 – 70.1) | -  0.01  0.02  0.04  <0.001 | -  0.3 (0.1 – 1.4)  0.5 (0.1 – 5.1)  0.4 (0.1 – 3.2)  0.4 (0.04 – 3.4) | -  0.12  0.57  0.40  0.38 |
| Work status   - Government employee - Non – government - Self employed - Non paid worker - Student - Home maker - Retired with benefits - Unemployed | 23 (6%)  16 (4%)  142 (34%)  29 (7%)  1 (0.2%)  18 (4%)  18 (4%)  167 (40%) | 1. (0.9 – 18)   4.7 (1.9 – 12)  7.9 (1.9 – 34)  -  -  -  6.7 (2.6 – 17) | -  0.07  <0.01  0.01  <0.001 | -  7.2 (1.2 – 42)  4.2 (1.2 – 15)  2.9 (0.4 – 21)  4.7 (1.2 – 18) | -  0.03*  0.03*  0.30  0.03* |
| Average earnings |  | 0.9 (0.9 – 0.9) | 0.03 | 1.0 (1.0 – 1.0) | 0.12 |
| Smoking   - Never smoked | 396 (96%) | 4 (1.5 – 10.8) | 0.01 | 0.5 (0.1 – 3.4) | 0.48 |
| Alcohol  - Never used | 398 (96%) | 4.9 (1.8 – 13.7) | <0.01 | 4.7 (0.7 – 32) | 0.12 |
| Body weight |  | 1.18 (1.0 – 1.04) | 0.09 | 1.04 (1.01 – 1.07) | 0.02* |

NB: Pearson χ2 goodness of fit p – value = 0.99; Hosmer – Lemeshow χ2 p – value = 0.53, (*) statistically significant
