# Supplementary material for: The prevalence of Schistosoma mansoni infection among adults with chronic non-communicable diseases in Malawi
Source: Trop Med Health. 2022 Aug 19;50:56. doi: 10.1186/s41182-022-00450-3 (PMC9389769; doi:10.1186/s41182-022-00450-3)
Supplement: Supplementary file 4 — Additional file 4: Regression analysis evaluating risk factors of diabetes mellitus. [file 41182_2022_450_MOESM4_ESM.docx]

Additional table S4. Regression analysis evaluating risk factors of diabetes mellitus

| Variable | N (%) | Crude OR  (95% CI) | P – value | Adjusted OR  (95% CI) | P – value |
| --- | --- | --- | --- | --- | --- |
| S. mansoni  - Positive | 50 (15%) | 0.6 (0.3 – 1.1) | 0.12 | - | - |
| Age |  | 0.96 (0.95 – 0.97) | <0.001 | 0.97 (0.95 – 0.98) | <0.001* |
| Sex   - Male - Female | 137 (33%)  277 (67%) | 1  0.3 (0.2 – 0.5) | <0.001 | 0.4 (0.2 – 0.7) | <0.01* |
| Education   - None - Less than primary - Primary school completed - Secondary school completed - College/University completed - Postgraduate degree | 88 (21%)  198 (48%)  56 (14%)  56 (14%)  11 (3%)  1 (1%) | 1   - 1. (0.9 – 2.7)   2. (0.9 – 3.8)   3. (2.1 – 8.6)  1. (2.3 – 56)   3.7 (0.6 – 24) | -  0.11  0.08  <0.001  <0.01  0.16 | -  1.2 (0.7 – 2.3)  1.3 (0.6 – 3.0)  2.1 (0.8 – 5.4)  3.5 (0.5 – 25)  1.3 (0.1 – 19) | -  0.50  0.48  0.12  0.22  0.87 |
| Marital status   - Never married - Currently married - Separated - Divorced - Widowed | 18 (4%)  248 (60%)  23 (6%)  31 (7%)  94 (23%) | 1  0.3 (0.1 – 0.8)  0.2 (0.04 – 0.6)  0.2 (0.1 – 0.8)  0.1 (0.02 – 0.3) | -  0.02  0.01  0.02  <0.001 | -  0.4 (0.1 – 2.3)  0.5 (0.1 – 3.0)  0.5 (0.1 – 2.9)  0.3 (0.1 – 1.8) | -  0.35  0.42  0.42  0.20 |
| Work status   - Government employee - Non – government - Self employed - Non paid worker - Student - Home maker - Retired with benefits - Unemployed | 23 (6%)  16 (4%)  142 (34%)  29 (7%)  1 (0.2%)  18 (4%)  18 (4%)  167 (40%) | 1   - 1. (0.2 – 3)   0.6 (0.2 – 1.5)  0.4 (0.1 – 1.2)  -  0.2 (0.05 – 0.7)  0.6 (5.4 – 2.2)  0.3 (0.1 – 0.8) | -  0.77  0.28  0.10  0.02  0.49  0.01 | -  0.7 (0.1 – 3.0)  0.97 (0.3 – 3.0)  0.6 (0.1 – 1.1)  0.5 (0.1 – 2.9)  0.1 (0.2 – 3.7)  0.02 (0.2 – 2.2) | -  0.60  0.95  0.51  0.47  0.80  0.52 |
| Average earnings |  | 1.0 (1 – 1.0) | 0.03 | 1.0 (1.0 – 1.0) | 0.95 |
| Smoking   - Never smoked | 396 (96%) | 1.9 (0.7 – 5.4) | 0.24 | 6.6 (1.2 – 35) | 0.03* |
| Alcohol  - Never used | 398 (96%) | 0.4 (0.1 – 1.1) | 0.09 | 0.6 (0.1 – 2.9) | 0.53 |
| Body weight |  | 1.01 (1.0 – 1.03) | 0.02 | 1.02 (1.00 – 1.03) | 0.03* |

NB: Pearson χ^2^ goodness of fit p – value = 0.06; Hosmer – Lemeshow χ^2^ p – value = 0.38; (*) statistically significant
